# Analysis of Lung Cancer Screening by Race After USPSTF Expansion of Screening Eligibility in 2021

**DOI:** 10.1001/jamanetworkopen.2022.17578

**Published:** 2022-06-15

**Authors:** Christine S. Shusted, Nathaniel R. Evans, Gregory C. Kane, Hee-Soon Juon, Julie A. Barta

**Affiliations:** 1 The Jane and Leonard Korman Respiratory Institute, Division of Pulmonary and Critical Care Medicine, Department of Medicine, Sidney Kimmel Medical College at Thomas Jefferson University, Philadelphia, Pennsylvania; 2 The Jane and Leonard Korman Respiratory Institute, Division of Thoracic Surgery, Department of Surgery, Sidney Kimmel Medical College at Thomas Jefferson University, Philadelphia, Pennsylvania; 3 Division of Population Science, Department of Medical Oncology, Sidney Kimmel Medical College at Thomas Jefferson University, Philadelphia, Pennsylvania

## Abstract

This cross-sectional study examines differences among individuals deemed eligible for lung cancer screening under USPSTF 2013 vs under USPTSF 2021 guidelines.

## Introduction

The US Preventive Services Taskforce (USPSTF) broadened lung cancer screening (LCS) eligibility in March 2021, lowering the minimum age to 50 years and decreasing smoking intensity to 20 pack-years.^1^ The expanded criteria have been projected to double the number of individuals with high risk eligible for LCS with low-dose computed tomography and further reduce lung cancer mortality.^2,3^ Moreover, the updated criteria may improve screening eligibility among vulnerable individuals, including African American, Hispanic, and female patients, and individuals who identify as members of the lesbian, gay, bisexual, transgender, queer, intersex, asexual communities as well as gender nonconforming individuals, who have previously experienced disproportionate underscreening.^3^ Specifically, African American individuals are less often eligible for LCS despite developing lung cancer at younger ages and with lower smoking intensity.^1^ Studies estimating the population-level outcomes associated with these changes in national cohorts have demonstrated mixed results with regard to relative increases in screening eligibility among racial and ethnic minorities, including African American individuals, and other underserved groups.^2,4,5^ There is a gap in knowledge about the directly observed impact of the USPSTF 2021 guidelines on LCS uptake in diverse populations. This study aims to begin to fill this gap by characterizing differences among individuals deemed eligible under USPSTF 2013 vs under USPTSF 2021 guidelines undergoing LCS in a centralized program.

## Methods

This cross-sectional study was approved by the Thomas Jefferson University institutional review board and granted a waiver of informed consent because this was a minimal risk, retrospective study of individuals already in our LCS Program Registry. We followed the Strengthening the Reporting of Observational Studies in Epidemiology (STROBE) reporting guideline.

Individuals who completed LCS between March 9 and December 9, 2021, through our centralized program at an urban, academic medical center were identified in our LCS Program Registry. This group was divided into a USPSTF 2013–eligible cohort (age ≥55 years and ≥30 pack-years, and quit <15 years ago) vs a USPSTF 2021–eligible cohort (age 50-54 years or 20-29 pack-years, and quit <15 years ago). Race, ethnicity, and gender were determined through self-report. For the purposes of this study examining race, race was recoded to African American, White, and other, which included individuals who identified as Alaskan Native or American Indian, Asian, Native Hawaiian or Pacific Islander, and more than 1 race. An historical cohort from the same 9-month period in 2019 was identified for additional comparison. Descriptive statistics, independent *t* tests, and χ^2^ tests were performed using a *P* < .05 significance threshold. Statistical analyses were 2-sided and conducted using SPSS statistical software version 26 (IBM) from December 13 to 16, 2021.

## Results

Baseline characteristics of the 815 individuals (mean [SD] age, 63.71 [5.98] years; 466 [57.2%] women) screened during the study period are displayed in the Table, including 161 patients (19.8%) who were newly eligible by USPSTF 2021 criteria and 654 individuals (80.2%) eligible under 2013 criteria. This USPSTF 2021–eligible cohort had a significantly greater proportion of African American individuals than the USPSTF 2013–eligible cohort (54.0% vs 39.5%; *P* = .002) (Table). Newly eligible individuals more frequently reported current smoking status (65.2% vs 55.0%; *P* = .02). As expected, individuals eligible under USPSTF 2021 criteria had a lower frequency of Medicare insurance and a significantly lower mean PLCOm2012 lung cancer risk (a lung cancer risk prediction model derived from the Prostate, Lung, Colorectal, and Ovarian Cancer Screening Trial and modified using National Lung Screening Trial data by Tammemagi and colleagues^6^) compared with the cohort eligible under USPSTF 2013 criteria. There was no significant difference in gender, distribution of educational attainment, or Lung Imaging Reporting and Data System (Lung-RADS) results between participants eligible under 2013 vs 2021 criteria. Comparison of the USPSTF 2013–eligible cohort screened in 2021 with a historical cohort screened in 2019 demonstrated no significant differences in age, gender, race or ethnicity, smoking history, PLCOm2012 risk, or Lung-RADS distribution.

**Table.** Baseline Characteristics of Individuals Undergoing Lung Cancer Screening Between March 9 and December 9, 2021

Characteristic	USPSTF eligibility criteria^a^		*P* value
	2021 (n = 161)	2013 (n = 654)	
Age, mean (SD), y	59.00 (6.02)	64.87 (5.37)	<.001
Gender			
Women	97 (60.2)	369 (56.4)	.38
Men	64 (39.8)	285 (43.6)	
Race			
African American	87 (54.0)	258 (39.5)	.002
White	64 (39.8)	357 (54.7)	
Other^b^	10 (6.2)	38 (5.8)	
Smoking status			
Current	105 (65.2)	360 (55.0)	.02
Former	56 (34.8)	294 (45.0)	
Pack-years, mean (SD)	36.86 (18.20)	52.40 (22.88)	<.001
Personal history of cancer	15 (9.3)	102 (15.8)	.11
Family history of lung cancer	42 (26.1)	175 (26.8)	.98
COPD	42 (26.1)	271 (41.4)	<.001
Education			
<High school diploma	24 (14.9)	85 (13.0)	.65
High school diploma/GED	70 (43.5)	289 (44.2)	
>High school diploma	64 (39.8)	256 (39.1)	
Unknown	3 (1.9)	24 (3.7)	
Insurance status			
Medicare	28 (17.4)	259 (39.6)	<.001
Medicaid or dual eligible	62 (38.5)	161 (24.6)	
Private	64 (39.8)	211 (32.3)	
Other or none	3 (2.6)	23 (3.5)	
PLCOm2012 lung cancer risk, mean (SD)^c^	3.27 (4.40)	6.64 (6.37)	<.001
Lung-RADS			
1	54 (33.5)	236 (36.1)	.79^d^
2	74 (46.0)	306 (46.8)	
3	17 (10.6)	55 (8.4)	
4A, 4B, 4X	10 (6.2)	41 (6.3)	
Not assigned	6 (3.7)	16 (2.4)	
Screen-detected lung cancer diagnosis	0	12 (1.8)	.08^d^

Abbreviations: COPD, chronic obstructive pulmonary disorder; GED, General Educational Development; Lung-RADS, Lung Imaging Reporting and Data System; USPSTF, US Preventive Services Task Force.

^a^ The USPSTF-eligible cohorts are comprised of individuals who were eligible and underwent lung cancer screening.

^b^ Includes individuals who self-identified as Alaskan Native or American Indian, Asian, Native Hawaiian or Pacific Islander, or more than 1 race.

^c^ The PLCOm2012 model is a lung cancer risk prediction model derived from the Prostate, Lung, Colorectal, and Ovarian Cancer Screening Trial and modified using National Lung Screening Trial data by Tammemagi and colleagues.^6^

^d^ Due to the small sample size in the USPSTF 2021–eligible cohort, the expected count for not assigned under Lung-RADS, and a lung cancer diagnosis under screen-detected lung cancer diagnosis was fewer than 5. Therefore, the statistical significance result from the χ^2^ test may not be valid.

## Discussion

This cross-sectional study of LCS uptake found that a significantly higher proportion of African American individuals were screened through our centralized LCS program after implementation of the expanded USPSTF 2021 criteria, but this was not observed among women or individuals with low educational attainment. Characterization of newly eligible and screened individuals is critical, as African American individuals, women, and other populations previously experiencing underscreening may in fact receive a greater lung cancer mortality benefit from LCS compared with White men.^2,3^ Although revision of the USPSTF criteria initially spurred optimism for more equitable screening, some population-based studies have noted that expanded eligibility may paradoxically serve to perpetuate racial, ethnic, and other disparities.^4,5^ LCS is a complex process with a multitude of potential barriers, and social determinants of health care, such as insurance status and access to care, may continue to disproportionately limit access to screening services for underserved populations.^4^

Limitations of this study include its single-institution design and a low rate of individuals reporting being a minority race or ethnicity other than African American. Future studies should examine USPSTF 2021–driven changes in LCS uptake as a function of relative changes in LCS eligibility to more completely define the impacts of the expanded criteria. Additional research is also needed in selection of LCS candidates using prediction models for lung cancer risk or life-years gained, which may improve screening efficiency for some groups, including those with lung cancer risk factors but who are ineligible for LCS.^5^

Expansion of screening criteria is a critical first step to achieving equity in LCS for all high-risk populations, but myriad challenges remain before individuals enter the door for screening. Health policy changes must occur simultaneously with efforts to expand community outreach, overcome logistical barriers, and facilitate screening adherence. Only after comprehensive strategies to dismantle screening barriers are identified, validated, and implemented can there be a truly equitable landscape for LCS.
